# Reproductive Benefit of Oxidative Damage: An Oxidative Stress “Malevolence”?

**DOI:** 10.1155/2011/760978

**Published:** 2011-09-28

**Authors:** B. Poljsak, I. Milisav, T. Lampe, I. Ostan

**Affiliations:** ^1^Laboratory for Oxidative Stress Research, Faculty of Health Sciences, University of Ljubljana, Zdravstvena Pot 5, 1000 Ljubljana, Slovenia; ^2^Institute of Pathophysiology, Faculty of Medicine, University of Ljubljana, 1000 Ljubljana, Slovenia; ^3^Faculty for Maritime Studies and Transportation, University of Ljubljana, 1000 Ljubljana, Slovenia

## Abstract

High levels of reactive oxygen species (ROS) compared to antioxidant defenses are considered to play a major role in diverse chronic age-related diseases and aging. Here we present an attempt to synthesize information about proximate oxidative processes in aging (relevant to free radical or oxidative damage hypotheses of aging) with an evolutionary scenario (credited here to Dawkins hypotheses) involving tradeoffs between the costs and benefits of oxidative stress to reproducing organisms. Oxidative stress may be considered a biological imperfection; therefore, the Dawkins' theory of imperfect adaptation of beings to environment was applied to the role of oxidative stress in processes like famine and infectious diseases and their consequences at the molecular level such as mutations and cell signaling. Arguments are presented that oxidative damage is not necessarily an evolutionary mistake but may be beneficial for reproduction; this may prevail over its harmfulness to health and longevity in evolution. Thus, Dawkins' principle of biological “malevolence” may be an additional biological paradigm for explaining the consequences of oxidative stress.

## 1. Introduction

The development of life on earth occurred alongside with the formation of free radicals (reviewed by [1, 2]). Free radicals are atoms, molecules, or ions with unpaired electrons in an open shell. Free radicals play a role in key biological processes such as cell division, cell decay, and death [1]. The cells of all present aerobic organisms produce the majority of chemical energy by consuming oxygen in their mitochondria, the main site of intracellular oxygen consumption and the main source of ROS formation. 

Mitochondrial sources are represented by the electron transport chain and the nitric oxide synthase reaction. The rate of mitochondrial respiration is responsible for the rate of generation of reactive oxygen species (ROS). Generally, the higher the metabolic rate of an organism, the shorter is its maximum lifespan; however, there are some exceptions to this rule [3, 4]. Estimates of how much oxygen reacts directly to generate free radicals vary; however, typically cited values are around 1.5–5% of the total consumed oxygen [5, 6]. These estimates have been questioned by Hansford et al. [7], and Staniek and Nohl [8], who suggested that H_2_O_2_ production rates were less than 1% of consumed O_2_. Yet, even if we accept a conservative value of 0.15%, this still represents a substantial amount of free radicals formation [9]. As mentioned, the rate of generation of H_2_O_2_ is dependent on the state of mitochondria as determined by the concentration of ADP, substrates, and oxygen [10]. 

Cells use antioxidants to neutralise ROS. The superoxide anion (O_2_^•^^−^), the direct product of mitochondrial metabolism, is neutralised by superoxide dismutase, producing hydrogen peroxide H_2_O_2_. This ROS is not very reactive; however, in the presence of some substances, it may trigger the formation of highly reactive free radicals; for instance, H_2_O_2_ is catalyzed by the free iron bivalent ions and leads to the generation of hydroxyl radical (OH•) in the Fenton reaction.

ROS may have a productive use as well. According to De Grey and Rae [11], evolution is an extremely clever engineer over the long term, which has learned ways of making the best of a bad job; for example, harnessing hydrogen peroxide for its own purposes [11]. The beneficial physiological cellular use of ROS is now being demonstrated in different fields, including intracellular signalling and redox regulation. Thus, our cells also generate some hydrogen peroxide deliberately for use as a chemical signal that regulates everything from glucose metabolism to cellular growth and proliferation [12]. The main synthesized ROS are superoxide radical and NO, which are produced by NADPH oxidases and NO synthases in different places of the organism [13]. These enzymes are highly active in most of the reproductive tissues, indicating that ROS are indeed necessary for reproduction. For example, a certain level of NO is necessary for mammalian spermatozoid maturation and activation [14]. The functioning of immune system, senses (sight) and other subsystems depends on use of ROS. The organisms have adapted their entire physiological machinery to our oxidative word. Oxidation allows obtaining energy for living and reproduction from diverse sources that were not available before the great oxidation event. 2,500 mya significant amounts of O_2_ appeared in the earth's atmosphere as a byproduct of the photosynthesis of blue-green algae, which enabled the development of anaerobic organisms (reviewed by [1, 2]). Thus, the problem is not the existence of ROS in living systems, but in the imbalance between ROS and antioxidants, that is, oxidative stress.

 Oxidative stress was first defined by Sies [15] as “a disturbance in the pro-oxidant-antioxidant balance in favour of the former, leading to potential damage.” Normal metabolism is associated with unavoidable mild oxidative stress resulting in biomolecular damage that cannot be totally repaired or removed by cellular degradative systems, including lysosomes, proteasomes, and cytosolic and mitochondrial proteases. Based on the amount of oxygen, altered nucleotides detected in human urine, it has been estimated that approximately 2 × 10^4^ oxidative DNA lesions occur per human genome every day [16]. The end result of these intrusions is impaired individual health. There is growing scientific evidence that oxidative stress is associated with chronic degenerative diseases like cancer, cardiovascular diseases, and diabetes and is considered an important factor in aging [1, 16]. 

Endogenous production of free radical and antioxidant defence systems in humans are very similar to those in many other mammals; however, there are animals with lesser mitochondrial production of free radicals and/or better antioxidant defence. For example, the idea that animal species that are exceptionally long lived for their body size (e.g., birds, turtles, bats, mole rats, etc.) are somehow resistant to oxidative damage is receiving a certain amount of attention by aging researchers. However, the nature of this resistance seems to be very complex, and a considerable amount of evidence addressing this premise to date has been equivocal. The question arises as to why evolution hasn't provided us (and many other animals) with such a seemingly beneficial phenotype. Some theories consider oxidative stress as a mistake or imperfection of basically “benevolent” biological processes “programmed” for preservation of life (homeostasis). To the contrary, some theories imply that at least some damaging effects of oxidative stress (like aging) are “programmed” to favour reproduction. The central concept of evolutionary senescence and life-history theory is the fact that oxidative stress is a normal byproduct of oxidative metabolism, while at the same time prooxidant molecules play crucial roles in normal cell signalling processes. This is a great example of an evolutionary scenario in which organisms have presumably been selected to trade off between the costs and benefits of essential but risky biochemical physiological processes (or genes with good early and bad late effects), and between somatic maintenance, growth, survival, life span, and reproduction. There are many aging theories, but none of the theories can explain the aging process in all the details, and some of the theories overlap. For example, mutation accumulation theory [17, 18] and antagonistic pleiotropy theory of aging [19] are not mutually exclusive, as both evolutionary mechanisms may operate at the same time. The main difference between the two theories is that in the mutation accumulation theory, genes with negative effects at old age accumulate passively from one generation to the next while, in the antagonistic pleiotropy theory, these genes are actively kept in the gene pool by selection [20]. Additionally, the disposable soma theory was proposed [21, 22], which postulated a special class of gene mutations with the following antagonistic pleiotropic effects; these hypothetical mutations save energy for reproduction (positive effect) by partially disabling molecular proofreading and other accuracy promoting devices in somatic cells (negative effect).

The evolutionary theory of aging is actually part of life history theory. Life history studies the changes organisms undergo from conception to death, but focuses particularly on the schedule of reproduction and survival [23, 24]. The evolutionary theories of aging are not ultimate completed theories, but rather a set of ideas that themselves require further elaboration and validation [25]. Additionally, there is a need to establish the link between ROS and reproductive aging in free-living animals [26]. 

The role of oxidative stress in the aging process, especially from an evolution point of view, will be discussed with an attempt to provide evidence or explanation of how oxidative stress can be seen as an imperfect adaptation of human evolution. A systematic confrontation of phenomena of oxidative stress against the basic theories on adaptation of beings to environment will be presented.

## 2. Theories on Adaptation of Beings to Environment

Two basic theories exist regarding the adaptation of beings to their environment. One of them is the theory of perfect adaptation of animals to the environment or “adaptationism,” defined as “that approach to evolutionary studies which assumes without further proof that all aspects of morphology, physiology, and behaviour of organisms are adaptive optimal solutions to problems” [27]. Some of the most representative adaptationists are Cain [28], Hamilton, Maynard, and Smith. 

The second basic approach is the theory of imperfect adaptation to the environment that treats “constraints on perfection” [29] as essential features of the biology of animals. Some arguments of Dawkins, the most prominent representative of this theory, are the following [29]:

- “time lags” (genes of present beings were selected in some earlier era when conditions were different),
- contradictory biology because of “historical constraints” (genes carry the heritage of adaptations to many types of past environments and might thus be contradictory),
- “mistakes due to environmental unpredictability” (organisms adapt to the “average” environment not to all environmental specifics at a given time),
- “constraints of costs and materials” (every evolutionary adaptation must cost something in lost opportunities to do other things and to adapt),
- biological “malevolence”; animals tend to maximise the survival (reproduction potential) of genes in peril even if that threatens to damage their own health.

Following the above list of Dawkins' arguments, the paper presents evidence and poses some unanswered questions regarding the imperfect nature of oxidative stress.

## 3. Questions regarding Problems of Time Lag, “Average Past Environment” and Contradictory Evolutionary Heritage

There is no doubt that the past evolutionary conditions shaped our present biology. However, beings need many generations to adapt to a new environment. One of the problems that this might cause is a time lag in adaptation to environmental changes. For instance, the quality of the air, especially its oxygen content has changed considerably during evolution [30, 31]. About 1,300 mya, the concentration of O_2_ in the atmosphere was only about 1%; 500 million years ago, it rose to about 10% and 5 million years ago reached the present level of about 21%. Currently, then, concentration of O_2_ in the air is higher than it was most of the time during the evolution of species. Have present day aerobes adapted perfectly to this high concentration of O_2_? How far back does our biological memory reach?

Antioxidant subsystems are very old (most part of the antioxidant system of humans is present at all the vertebrate systems) and developed early in the evolution, most probably together with increased concentration of oxygen in the atmosphere. The principles of cell defence against oxidative stress, for example, the nature and role of antioxidants and antioxidative enzymes acting to decrease ROS concentrations, the repair of damaged macromolecules, and the elimination of irreparable proteins are basically similar at all levels of cell organization [32]. One of the oldest antioxidants, common to all animals and plants, is melatonin, which evolution probably introduced shortly after the appearance of the first photosynthetic bacteria [33]. Mitochondria are much younger, but still very old and a very conservative cell subsystem as well. 

All these biological subsystems had to adapt to ever changing food, air, and other environmental conditions. The increase of oxygen concentration in the air, for example, experienced long periods of major oscillations that might be an additional source of problems of biological adaptation. During most of the Palaeozoic, its concentration didn't reach 15%, but, in the Carboniferous era, it rose sharply, reaching a peak of about 35% (286 mya), falling again below 20% during the early Mesozoic and rising again to about 25% at the beginning of the Tertiary (60 mya), to fall gradually to the present atmospheric level of 21% [1, 2]. Which is then the “average past environment” that our most conservative antioxidant subsystems and mitochondrial processes have mostly adapted to?

## 4. “Costs and Materials”

One of important causes of imperfect defence from ROS damage is its high cost in terms of energy expenditures. The body uses energy from food for metabolism, reproduction, repair, and maintenance. Thus, mechanisms that protect cells from oxidative stress (e.g., endogenous antioxidants and DNA repair processes) are consuming significant amounts of energy when being activated in all compartments of a cell all of the time. It may require too much energy to build enough defences to prevent all oxidative damage all the time throughout the life of an organism. Kowald and Kirkwood [34, 35] proposed a quantitative MARS (mitochondria, aberrant proteins, radicals, and scavengers) model. Using this simulation, they predicted that virtual immortality might be achieved if 55% of the total energy of the simulated cell was devoted to repair and/or prevention of free-radical and oxidative damage. It is the compromise in allocating (less) energy to the repair mechanisms that causes the body to deteriorate gradually with age [21]. Limited food supply influences lifespan. Among mammals, the long-living species are generally those that are most highly evolved and therefore possess the most sophisticated mechanisms for competing for food although exceptions could be found like longevous trees and apparently immortal bacteria. Let's explain the problem of costly defence from ROS using examples of aging problems and infections.

### 4.1. Aging

Nature is a highly competitive place and almost all animals in the wild die before they attain old age and their maximum lifespan [36]. For example, 90% of wild mice are dead by the age of 10 months although the same animals might live for three years in a protective environment [37, 38]. If 90% of wild mice die within 10 months, any investment in maintenance to keep the body in good condition much beyond this point is beneficial for 10% of the population at most. This immediately implies that there will be little evolutionary advantage in building long-term survival capacity into a mouse [36]. The argument is further strengthened when we observe that nearly all of the survival mechanisms required by the mouse to combat intrinsic deterioration (e.g., oxidative damage) require metabolic resources. These are scarce, as is evidenced by the fact that the major cause of mortality for wild mice is lack of food and hypothermia, due to insufficient energy to maintain body temperature [39]. From a Darwinian point of view, the mouse will benefit more from investing any spare resources into thermogenesis or reproduction than into improvement of DNA repair capacity necessary to ensure adequate function for a limited time period [36]. The fact that endogenous antioxidant defences and repair systems are not automatically present all the time but must be induced by elevated ROS formation suggests that they are indeed costly from energetic point of view.

Therefore, there is no evolutionary drive to keep the body fit for the long haul—not much selection pressure for traits that would maintain viability past the time when most animals would most likely be dead, killed by predators or disease, accident, coldness, or famine. According to some theorists, long postreproductive lifespan is selected against because such aged individuals consume food resources while not contributing further to the gene pool [40, 41]. Evolutionary processes evolved to favour investing more energy in the germline at the expense of the soma. Human germ cells have arguably lived for millions of years through an investment in DNA-repair enzymes, antioxidant enzymes, and telomerase [42]. The allocation of more energy to the germline allows us to reproduce more effectively, transferring DNA to our children with more accuracy and with lower frequency of lethal genetic aberrations. It also allows us to reproduce longer; the germline maintains its integrity for longer periods, and we have healthier children later in life. From this perspective, having the somatic cells that protect the germline periodically replaced is a small price to pay for the evolutionary advantages that drastically increase fitness [43]. In general, when a species has fewer predators, evolution invests fewer resources into speedy reproduction and more into genetic resources (DNA repair, etc.), that is, into a longer reproductive period (longer life).

### 4.2. Infections

Chronic inflammation is a source of cellular damage. When an infection occurs, immune cells secrete large amounts of free radicals to combat the invader. But these inflammatory chemicals also attack normal tissue surrounding the infection and damage critical components of cells, including DNA. During chronic inflammation, that damage may lead to mutations or cell death and even to cancer and other diseases [44–48].

Thus, a better cell antioxidant protection system would favour a quicker recovery in cases of inflammation, but in evolution it hasn't developed probably because of the necessary high expenditure in terms of costs and materials that are always limited. Besides, innate, inflammation-based immunity is the first line of vertebrate defence against microorganisms. Inflammation relies on a number of cellular and molecular effectors that can strike invading pathogens very shortly after the encounter between inflammatory cells and the intruder, but in a nonspecific way. Owing to this nonspecific response, inflammation can generate substantial costs for the host if the inflammatory response and the associated oxygen-based damage get out of control [49].

## 5. “Malevolence”—Programmed Damaging of Individual Health and Longevity in Favour of Gene (Species) Reproduction

These views of oxidative damage might be considered as “benevolent” imperfections, they are mistakes, or “malus minor”, in biological functioning that tend to reach individuals best (including longevity), but constraints in living conditions prevent reaching it in a perfect way. The principle of “malevolence” means that individual's biology doesn't tend toward longevity, but rather reproduction and therefore health and longevity damage are biologically programmed. The first to use the term “malevolence” in similar sense was Dawkins as a part of his theory of imperfect adaptation to environment, referring to the principle of “mistakes due to environmental unpredictability or “malevolence”.

The duplication is successful if the copies are the same as the original (the principle of fidelity) and if they are numerous (the principle of fecundity) [29]. Conditions required for multiplication are usually different from those needed for health and reproduction and usually prevail over survival. As the body is just a vehicle for gene multiplication [50], the soma is programmed to function for efficient reproduction and to be sacrificed when it is not useful for this purpose. According to Metcalfe and Alonso-Alvarez [26], oxidative stress may both stimulate and be caused by reproduction.

Biological “malevolence” might be found not only on the physiological level but on the behavioural (instinctive) as well.

### 5.1. Malevolent Instinctive Behaviour

In biology, it is well known that the desire for multiplication is the strongest instinct in nature [51, 52]. However nutritional instinct seems to be mostly regulated by reproductive needs as well (see [53, 54]). Here we briefly review some nutritional cases of malevolent instinctive drives from the aspect of oxidative damage:

#### 5.1.1. Instinctive Repulsion of Caloric Restriction

There is a growing corpus of research evidence that caloric restriction reduces the mitochondrial production of free radicals (as the most important internal source of reactive oxygen species) and enhances the antioxidant defence of organisms [1, 2, 55]. Tests performed on different species, from unicellular organisms to primates, have shown that a reduction of 30 to 50% of caloric intake (whilst retaining the intake of essential nutrients) extends the lifespan by about 30 to 50% and also improves health [56, 57]. Concurrently, the same reduction of caloric intake reduces fertility and causes retardation of sexual maturation [56–58] as sexuality and other reproductive function are energetically demanding processes. 

Hypothetically, a being could choose nutrition suitable for longevity or for reproduction. However, instinctive drives prevent animals from choosing that caloric restricted nutrition that is best for their longevity and leads them to consume an energy-rich diet. Restrictions on caloric intake produce stress hormones [40, 41] and rather intense discomfort of hunger.

 In the previous section, we presented arguments of some researchers who claim that a long postreproductive lifespan is selected against because such aged individuals consume food resources although, for example, old women could contribute by maternal care on grandchildren, which could partially explain differences in longevity between sexes in the human beings. This explanation is weakened by research results showing that a longer lifespan could be achieved with the same amount of food without minor reproductive success. Research on middle aged and old animals also shows that caloric restriction boosts their health and longevity [40, 41] although, again, some authors defend that the beneficial effects of caloric restriction mostly act in young developing individuals (e.g. [59]). Thus, nature could evolve instinctive preference of old subjects for a caloric restricted diet achieving a longer lifespan with the same total amount of food consumption (eating less food daily for a longer period). Instead, it also pushes old individuals to a self-destructive caloric rich diet.

#### 5.1.2. Instinctive Drive for Oxidising Nutritional Patterns

Other instinctive nutritional drives like attractiveness of food stimulants and cooked food [53, 54] also seem to be “malevolent.” Smoking [60] and alcohol abuse [61] are strong sources of oxidative stress. The final result of consumption of drugs is depletion of body energy resources [62, 63]. However, all animals search for food stimulants [64, 65]. The inclination of humans towards alcohol consumption is not a result of prevalent past nutritional practice [66]; this seems to be a more permanent innate preference of different species.

The oxidising food induces a quick transformation into blood sugar [62], which is useful for energy-demanding reproductive functions [54]. It is well known that heat treatment of food destroys some important antioxidants (like vitamin C and E). It also causes the transformations of some nutrients into mutagenic and carcinogenic oxidants [54]. However, many wild animals, including great apes, prefer cooked food to raw [66]. Cooking speeds up the absorption of energy from food, which is beneficial for reproduction and thus is instinctively preferred despite health damage in the long run [54].

### 5.2. Malevolent Physiology

#### 5.2.1. Mutations

An example of malevolent event might be a *mutation*. Most mutations are harmful. From the individual point of view, they cause damage or are not useful at best. However, in view of gene reproduction in a dynamic environment, they are very useful. Once in a while, a mutation offers a survival and reproductive advantage in a given environment [67]. In humans, spermatozoa frequently possesses high level of nuclear damage [68]. Several studies in vertebrates indicate that the mutation rate is higher in spermatogenesis than in oogenesis [69–71]. Velando et al. [72] proposed the hypothesis that the oxidation of DNA in the germ line is a prominent force in the evolution of mate choice and sexual signalling. The hypothesis assumes that, by avoiding oxidatively damaged sperm, the choosy sex avoids heritable effects derived from oxidative DNA damage.

There are species which reduce oxidative stress by stabilizing mitochondrial structure and energy efficiency. Exceptionally long-living mammals and birds have a more peroxidation-resistant membrane composition compared to shorter-living similar-sized mammals [73, 74]. Saturated and monounsaturated fatty acids are resistant to peroxidative damage while the more polyunsaturated a fatty acid is, the more susceptible it is to peroxidation. In the case of birds, the mitochondrial membranes show lower levels of fatty acid unsaturation, mainly due to a substitution of highly unsaturated fatty acids by linoleic acid, making these mitochondria more resistant to lipid peroxidation. Additionally, the protein complexes of the respiratory chain of mitochondria generate fewer free radicals in birds [73, 74]. Birds thus have a much higher maximum longevity than mammals of similar metabolic rate [73, 74]. There are examples of species that have a much more efficient DNA repair system than others. Species like Blanding's turtle (*Emydoidea blandingii*) and painted turtle (*Chrysemys picta*) have not shown signs of aging in studies lasting decades [75, 76]. This phenomenon seems to occur due to the uniqueness of telomere biology in turtles [77], and some evidence suggests that the cells of turtles have enhanced mechanisms to protect against reactive oxygen species formation and damage [78]. Krivoruchko and Storey [79] suggested other mechanism, that is anoxia tolerance, which includes metabolic rate depression, strong antioxidant defenses, activation of specific stress-responsive transcription factors, and enhanced expression of cyto-protective proteins. However, this might be an obstacle for their evolution. Some species of turtles have remained the same since the era of dinosaurs. 

The intensity and incidence of aging appears to be higher in mammals than in reptiles. A careful analysis of the aging phenotype of mammals and reptiles reveals an extraordinary contrast [80]. For example, reproductive senescence, in the form of no oocyte regeneration, is thought to occur in all studied mammals, but not in reptiles. Continuous tooth development is another common feature of reptiles absent from nearly all mammals. Therefore, some researchers have found it bizarre that all studied mammals feature aging when more primitive species such as fish and reptiles appear to avoid it [81].

In a dynamic environment, DNA-oxidative damage is beneficial for genetic pool reproduction, providing genetic diversity that is a necessary condition of group survival in such conditions [82]. 

#### 5.2.2. Senescence Disruption of Cell Signalling

A possible case of “malevolent” physiological change is the senescence decay of cell signalling based on ROS.

ROS induce various biological processes that include a transient elevation of intracellular Ca^2+^ concentration, phosphorylation of specific proteins, activation of specific transcription factors, modulation of eicosanoid metabolism, and stimulation of cell growth [10]. Nitric oxide was identified as a signalling molecule as early as 1987 [83] and is now a well-known regulator of some transcription factor activities and other determinants of gene expression. Hydrogen peroxide and superoxide have similar intracellular effects [84]. ROS can affect directly conformation and/or activities of all sulfhydryl-containing molecules, such as proteins or glutathione (GSH), by oxidation of their thiol moiety. Among many other enzymes and membrane receptors, this type of redox regulation affects many proteins important in signal transduction and carcinogenesis, such as protein kinase C, Ca^2+^-ATPase, collagenase, and tyrosine kinase [85]. For several transcription factors, ROS are physiological mediators of transcription control [86]. The well-known examples of redox-sensitive transcription factors are nuclear factor-*κ*B (NF-*κ*B) and activator protein-1 (AP-1). Thus, increased oxidative stress is not beneficial for the cell; however, an increase in cellular antioxidants might impact redox regulation and signal transduction. The complete elimination of free radicals would thus disrupt, rather than extend, the normal functioning of the body.

To control these reactive species with “two faces,” cells evolved complex and critical regulatory mechanisms which become disrupted with age [87]. Senescence is just an example of the pathophysiological implications of redox dysregulations [88]. The initiation of aging is marked by a shift from redox regulation to redox dysregulation [58]. Why this shift takes place is not yet clear. This could be considered as a biological “malevolence” towards aged individuals that are mostly useless for achieving the primary evolutionary goal—genes reproduction.

## 6. Conclusions

The paper presents some explanations for oxidative stress phenomena applying the field of Dawkins' theory of imperfect adaptation of beings to the environment. In this theory, the principle of “malevolence” plays a central role, and, according to Dawkins, there are objective historical constraints for perfect adaptation to environment (the problems of evolutionary “time lag,” contradictory heritage from different past evolutionary eras, constraints in cost and materials…); however, the criterion of adaptation of beings to environment is reproductive success, not longevity. Health is supported as long it is needed for reproduction. Many studies have been devoted to explore the different perspectives on the role of oxidative stress as constraint in life-history evolution [26, 89, 90]. In numerous species, it has been observed that longevity is negatively correlated with reproduction, but the physiological basis of this cost is not well understood. The study of Salmon et al. [91] on fruit flies suggests that oxidative stress susceptibility is associated with increased egg production in Drosophila melanogaster and is thus a physiological cost of reproduction. Additionally, Alonso-Alvarez et al. [92, 93] showed that male Zebra finches treated with testosterone had a reduced ability to fight free radicals [94]. Results suggest that only high-quality males should be able to afford the oxidative challenge promoted by testosterone [94].

Our review expands the earlier ideas of the disposable soma theory. We additionally suggest that oxidative stress is the basic mechanism of disposable soma theory. The review presents some additional evidence of the malevolent nature of oxidative stress phenomena. Senescence is the subproduct of this evolutionary strategy leading to increaseing individual Darwinian fitness. Considering the central role of this principle in Dawkins theory raises the question: Might the reproductive benefit of oxidative damage be viewed as a general cause of oxidative stress phenomena? Is such a “theory of oxidative stress malevolence” a correct explanation for this type of biological damage? Could we conclude that we oxidize to reproduce? 

In the end, we would like to stress the quote by Gavrilov and Gavrilova [25] about the evolutionary theories of aging: “They are useful when they open new opportunities for research by suggesting testable predictions, but they should never be used to impose limitations on aging studies. This is because the evolutionary “theories” of aging are not completed theories, but rather a set of ideas that require further elaboration and validation.” Thus, the integration of oxidative stress, into theories of aging and evolution is still at its beginning. There is a need for a general explanation of causes for oxidative stress and for this reason the aim of this paper is to stimulate the scientific discussion and further research.

## References

[1] Halliwel B, Gutteridge JMC (2005). *Free Radicals in Biology and Medicine*.

[2] Halliwel B, Gutteridge J (1999). *Free Radicals in Biology and Medicine*.

[3] Sohal RS, Witler R (1976). Metabolic rate and life span. *Cellular Aging: Concepts and Metabolism*.

[4] Sohal RS, Mockett RJ, Orr WC (2002). Mechanisms of aging: an appraisal of the oxidative stress hypothesis. *Free Radical Biology and Medicine*.

[5] Beckman KB, Ames BN (1998). The free radical theory of aging matures. *Physiological Reviews*.

[6] Casteilla L, Rigoulet M, Penicaud L (2002). Mitochondrial ROS metabolism: modulation by uncoupling proteins. *IUBMB Life*.

[7] Hansford RG, Hogue BA, Mildaziene V (1997). Dependence of H_2_O_2_ formation by rat heart mitochondria on substrate availability and donor age. *Journal of Bioenergetics and Biomembranes*.

[8] Staniek K, Nohl H (1999). H(2)O(2) detection from intact mitochondria as a measure for one-electron reduction of dioxygen requires a non-invasive assay system. *Biochimica et Biophysica Acta*.

[9] Speakman JR, Selman C, McLaren JS, Harper EJ (2002). Living fast, dying when? The link between aging and energetics. *Journal of Nutrition*.

[10] Kaul N, Forman HJ, Rhodes C (2000). Reactive oxygen species in physiology and toxicology: from lipid peroxidation to transcriptional activation. *Toxicology of the Human Environment: The Critical Role of Free Radicals*.

[11] De Grey A, Rae M (2007). *Ending Aging*.

[12] Rhee SG (1999). Redox signaling: hydrogen peroxide as intracellular messenger. *Experimental and Molecular Medicine*.

[13] Bedard K, Krause KH (2007). The NOX family of ROS-generating NADPH oxidases: physiology and pathophysiology. *Physiological Reviews*.

[14] Revelli A, Ghigo D, Moffa F, Massobrio M, Tur-Kaspa I (2002). Guanylate cyclase activity and sperm function. *Endocrine Reviews*.

[15] Sies H (1991). *Oxidative Stress II. Oxidants and Antioxidants*.

[16] Ames BN, Shigenaga MK, Hagen TM (1993). Oxidants, antioxidants, and the degenerative diseases of aging. *Proceedings of the National Academy of Sciences of the United States of America*.

[17] Medawar PB (1946). Old age and natural death. *Modern Quarterly*.

[18] Medawar PB (1952). *An Unsolved Problem of Biology*.

[19] Williams GC (1957). Pleiotropy, natural selection and the evolution of senescence. *Evolution*.

[20] Le Bourg É (2001). A mini-review of the evolutionary theories of aging. Is it the time to accept them?. *Demographic Research*.

[21] Kirkwood TBL (1977). Evolution of ageing. *Nature*.

[22] Kirkwood TBL, Holliday R (1979). The evolution of ageing and longevity. *Proceedings of the Royal Society of London B*.

[23] Stearns SC (1992). *The Evolution of Life Histories*.

[24] Charnov EL (1993). *Life History Invariants: Some Explorations of Symmetry in Evolutionary Ecology*.

[25] Gavrilov LA, Gavrilova NS (2002). Evolutionary theories of aging and longevity. *TheScientificWorldJournal*.

[26] Metcalfe NB, Alonso-Alvarez C (2010). Oxidative stress as a life-history constraint: the role of reactive oxygen species in shaping phenotypes from conception to death. *Functional Ecology*.

[27] Lewontin RC (1979). Sociobiology as an adaptationist program. *Behavioral Science*.

[28] Cain AJ (1979). Introduction to general discussion. Evolution of adaptation by natural selection. *Proceedings of the Royal Society of London B*.

[29] Dawkins R (1982–1999). *The Extended Phenotype: The long Reach of the Gene*.

[30] Balantine J (1982). *Pathology of Oxygen Toxicity*.

[31] Gilbert DL (1981). *Oxygen and Living Processes: An Interdisciplinary Approach*.

[32] Sigler K, Chaloupka J, Brozmanova J, Stadler N, Hofer M (1999). Oxidative stress in microorganisms. *Folia Microbiologica*.

[33] Russel JR, Robinson J (1995). *Melatonin.*.

[34] Kowald A, Kirkwood TBL (1994). Towards a network theory of ageing: a model combining the free radical theory and the protein error theory. *Journal of Theoretical Biology*.

[35] Kowald A, Kirkwood TBL (1996). A network theory of ageing: the interactions of defective mitochondria, aberrant proteins, free radicals and scavengers in the ageing process. *Mutation Research*.

[36] Kirkwood B, Mathers JC, Stanner S, Thompson R, Buttriss J (2009). The basic biology of aging. *Healthy Aging—The Role of Nutrition and Lifestyle*.

[37] Austad SN (1997). Comparative aging and life histories in mammals. *Experimental Gerontology*.

[38] Austad SN (1997). *Why We Age: What Science Is Discovering about the Body’s Journey through Life*.

[39] Berry RJ, Bronson FH (1992). Life-history and bioeconomy of the house mouse. *Biological Reviews of the Cambridge Philosophical Society*.

[40] Mattson MP, Duan W, Wan R, Guo Z, Mattson MP (2003). Cellular and molecular mechanisms whereby dietary restriction extends healthspan: a beneficial type of stress. *Energy Metabolism and Lifespan Determination*.

[41] Mattson MP, Duan W, Wan R, Guo Z (2003). Cellular and molecular mechanisms whereby dietary restriction extends healthspan: a beneficial type of stress. *Advances in Cell Aging and Gerontology*.

[42] Best B Mechanisms of aging. http://www.benbest.com/lifeext/aging.html.

[43] Smelick  C (2003). http://www.biologicalgerontology.com.

[44] Parsonnet J (1995). Bacterial infection as a cause of cancer. *Environmental Health Perspectives*.

[45] Ohshima H, Tatemichi M, Sawa T (2003). Chemical basis of inflammation-induced carcinogenesis. *Archives of Biochemistry and Biophysics*.

[46] Macarthur M, Hold GL, El-Omar EM (2004). Inflammation and Cancer II. Role of chronic inflammation and cytokine gene polymorphisms in the pathogenesis of gastrointestinal malignancy. *American Journal of Physiology*.

[47] Federico A, Morgillo F, Tuccillo C, Ciardiello F, Loguercio C (2007). Chronic inflammation and oxidative stress in human carcinogenesis. *International Journal of Cancer*.

[48] Kundu JK, Surh YJ (2008). Inflammation: gearing the journey to cancer. *Mutation Research*.

[49] Sorci G, Faivre B (2009). Inflammation and oxidative stress in vertebrate host-parasite systems. *Philosophical Transactions of the Royal Society B*.

[50] Dawkins R (1976). *The Selfish Gene/ Sebični Gen*.

[51] Bryson B (2003). *A Short History of Nearly Everything*.

[52] Ridley M (1999). Genome: the autobiography of a species. *Genom: Biografija človeške vrste*.

[53] Ostan I, Poljšak B, Simčič M, Tijskens LMM (2010). Appetite for the selfish gene. *Appetite*.

[54] Ostan I, Poljšak B, Simčič M, Tijskens LMM (2009). Nutrition for the selfish gene. *Trends in Food Science & Technology*.

[55] Poljsak B (2010). *Decreasing Oxidative Stress and Retarding the Aging Process*.

[56] Gredilla R, Barja G, Mattson MP (2003). Mitochondrial oxidative stress and caloric restriction. *Energy Metabolism and Lifespan Determination*.

[57] Gredilla R, Barja G (2003). Mitochondrial oxidative stress and caloric restriction. *Advances in Cell Aging and Gerontology*.

[58] Humphries KM, Szweda PA, Szweda LI (2006). Aging: a shift from redox regulation to oxidative damage. *Free Radical Research*.

[59] Lipman RD, Smith DE, Bronson RT, Blumberg J (1995). Is late-life caloric restriction beneficial?. *Aging (Milano)*.

[60] Wooten J, Chouchane S, McGrath TE, Haliwell BB, Poulsen HE (2006). Tobacco smoke constituents affecting oxidative stress. *Cigarette Smoke and Oxidative Stress*.

[61] Loguercio C, Tuccillo C, Federico A, Fogliano V, Del Vecchio Blanco C, Romano M (2009). Alcoholic beverages and gastric epithelial cell viability: effect on oxidative stress-induced damage. *Journal of physiology and pharmacology*.

[62] Holford P (2004). *Patrick Holford’s New Optimum Nutrition Bible*.

[63] Ichazo O, Holford P (2004). The African. *Patrick Holford’s New Optimum Nutrition Bible*.

[64] Engel C (2002). *Wild Health: How Animals Keep Themselves Well and What We Can Learn from Them*.

[65] Rafert J, Vineberg EO (1997). Bonobo nutrition—relation of captive diet to wild diet. *Bonobo Husbandry Manual*.

[66] Wobber V, Hare B, Wrangham R (2008). Great apes prefer cooked food. *Journal of Human Evolution*.

[67] Olshansky SJ, Carnes BA (2001). *Science at the Frontiers of Aging. The Guest for Immortality*.

[68] Migliore L, Colognato R, Naccarati A, Bergamaschi E (2006). Relationship between genotoxicity biomarkers in somatic and germ cells: findings from a biomonitoring study. *Mutagenesis*.

[69] Carmichael AN, Fridolfsson AK, Halverson J, Ellegren H (2000). Male-biased mutation rates revealed from Z and W chromosome-linked ATP synthase alpha-subunit (ATP5A1) sequences in birds. *Journal of Molecular Evolution*.

[70] Zhang J (2004). Evolution of DMY, a newly emergent male sexdetermination gene of medaka fish. *Genetics*.

[71] Taylor J, Tyekucheva S, Zody M, Chiaromonte F, Makova KD (2006). Strong and weak male mutation bias at different sites in the primate genomes: insights from the human-chimpanzee comparison. *Molecular Biology and Evolution*.

[72] Velando A, Torres R, Alonso-Alvarez C (2008). Avoiding bad genes: oxidatively damaged DNA in germ line and mate choice. *BioEssays*.

[73] Hulbert AJ (2008). Explaining longevity of different animals: is membrane fatty acid composition the missing link?. *Age*.

[74] Hulbert AJ, Pamplona R, Buffenstein R, Buttemer WA (2007). Life and death: metabolic rate, membrane composition, and life span of animals. *Physiological Reviews*.

[75] Congdon JD, Nagle RD, Kinney OM, van Loben Sels RC (2001). Hypotheses of aging in a long-lived vertebrate, Blanding’s turtle (Emydoidea blandingii). *Experimental Gerontology*.

[76] Congdon JD, Nagle RD, Kinney OM, van Loben Sels RC, Quinter T, Tinkle DW (2003). Testing hypotheses of aging in long-lived painted turtles (Chrysemys picta). *Experimental Gerontology*.

[77] Girondot M, Garcia J, Miaud C, Guyétant R Senescence and longevity in turtles: what telomeres tell us.

[78] Lutz PL, Prentice HM, Milton SL (2003). Is turtle longevity linked to enhanced mechanisms for surviving brain anoxia and reoxygenation?. *Experimental Gerontology*.

[79] Krivoruchko A, Storey KB (2010). Forever young: mechanisms of natural anoxia tolerance and potential links to longevity. *Oxidative Medicine and Cellular Longevity*.

[80] Comfort A http://www.senescence.info/evolution.html.

[81] Hayflick L (1994). *How and Why We Age*.

[82] Hawks J, Wang ET, Cochran GM, Harpending HC, Moyzis RK (2007). Recent acceleration of human adaptive evolution. *Proceedings of the National Academy of Sciences of the United States of America*.

[83] Palmer RMJ, Ferrige AG, Moncada S (1987). Nitric oxide release accounts for the biological activity of endothelium-derived relaxing factor. *Nature*.

[84] Kamata H, Hirata H (1999). Redox regulation of cellular signalling. *Cellular Signalling*.

[85] Dalton TP, Shertzer HG, Puga A (1999). Regulation of gene expression by reactive oxygen. *Annual Review of Pharmacology and Toxicology*.

[86] Nordberg J, Arner ESJ (2001). Reactive oxygen species, antioxidants, and the mammalian thioredoxin system. *Free Radical Biology and Medicine*.

[87] Gilca M, Stoian I, Atanasiu V, Virgolici B (2007). The oxidative hypothesis of senescence. *Journal of Postgraduate Medicine*.

[88] Valko M, Leibfritz D, Moncol J, Cronin MTD, Mazur M, Telser J (2007). Free radicals and antioxidants in normal physiological functions and human disease. *International Journal of Biochemistry and Cell Biology*.

[89] Dowling DK, Simmons LW (2009). Reactive oxygen species as universal constraints in life-history evolution. *Proceedings of the Royal Society B: Biological Sciences*.

[90] Monaghan P, Metcalfe NB, Torres R (2009). Oxidative stress as a mediator of life history trade-offs: mechanisms, measurements and interpretation. *Ecology Letters*.

[91] Salmon AB, Marx DB, Harshman LG (2001). A cost of reproduction in Drosophila melanogaster: stress susceptibility. *Evolution*.

[92] Alonso-Alvarez C, Bertrand S, Devevey G (2004). An experimental test of the dose-dependent effect of carotenoids and immune activation on sexual signals and antioxidant activity. *American Naturalist*.

[93] Alonso-Alvarez C, Bertrand S, Devevey G (2006). An experimental manipulation of life-history trajectories and resistance to oxidative stress. *Evolution*.

[94] Alonso-Alvarez C, Bertrand S, Faivre B, Chastel O, Sorci G (2007). Testosterone and oxidative stress: the oxidation handicap hypothesis. *Proceedings of the Royal Society B*.

